# Dipolar repulsion in α-halocarbonyl compounds revisited†

**DOI:** 10.1039/d1cp02502c

**Published:** 2021-09-01

**Authors:** Daniela Rodrigues Silva, Lucas de Azevedo Santos, Trevor A. Hamlin, F. Matthias Bickelhaupt, Matheus P. Freitas, Célia Fonseca Guerra

**Affiliations:** Department of Theoretical Chemistry, Amsterdam Institute of Molecular and Life Sciences (AIMMS), Amsterdam Center for Multiscale Modeling (ACMM), Vrije Universiteit Amsterdam De Boelelaan 1083 1081 HV Amsterdam The Netherlands f.m.bickelhaupt@vu.nl c.fonsecaguerra@vu.nl; Departamento de Química, Instituto de Ciências Naturais, Universidade Federal de Lavras 37200-900 Lavras MG Brazil matheus@ufla.br; Institute for Molecules and Materials (IMM), Radboud University Heyendaalseweg 135 6525 AJ Nijmegen The Netherlands; Leiden Institute of Chemistry, Gorlaeus Laboratories, Leiden University Einsteinweg 55 2333 CC Leiden The Netherlands

## Abstract

The concept of dipolar repulsion has been widely used to explain several phenomena in organic chemistry, including the conformational preferences of carbonyl compounds. This model, in which atoms and bonds are viewed as point charges and dipole moment vectors, respectively, is however oversimplified. To provide a causal model rooted in quantitative molecular orbital theory, we have analyzed the rotational isomerism of haloacetaldehydes OHC–CH_2_X (X = F, Cl, Br, I), using relativistic density functional theory. We have found that the overall trend in the rotational energy profiles is set by the combined effects of Pauli repulsion (introducing a barrier around *gauche* that separates minima at *syn* and *anti*), orbital interactions (which can pull the *anti* minimum towards *anticlinal* to maximize hyperconjugation), and electrostatic interactions. Only for X = F, not for X = Cl–I, electrostatic interactions push the preference from *syn* to *anti*. Our bonding analyses show how this trend is related to the compact nature of F *versus* the more diffuse nature of the heavier halogens.

## Abstract 2

Beyond point charges! The point charge concept within dipolar repulsion model is valid for compact atoms like fluorine. This model breaks down for larger halogens, for which the electrostatic attraction between nuclei and charge densities dominates.

## Graphical abstract

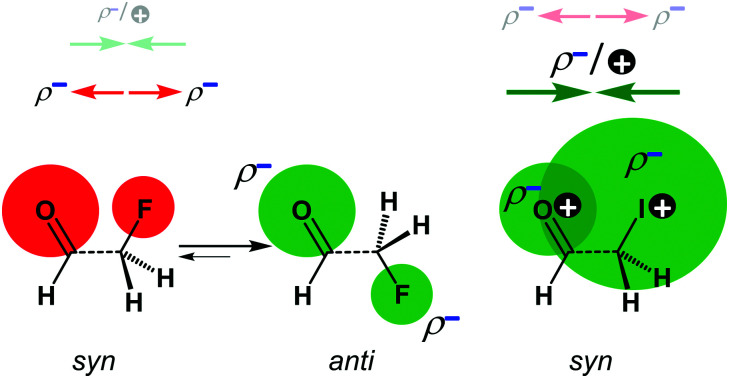

## Introduction

The carbonyl group is one of the most common functional groups in organic chemistry. Its unique chemistry renders the carbonyl group a site for a wide spectrum of chemical transformations^1^ and interactions^2^ that govern the structure of important biological systems, such as proteins,^3^ and nucleic acids.^4^ Attempts to understand and rationalize the structure and properties of compounds bearing a carbonyl group abound.^5^ In particular, the forces underlying their conformational preferences, and the resulting influence on their physical, chemical, and biological properties, have intrigued organic chemists for decades.^6^

**Fig. 1:**
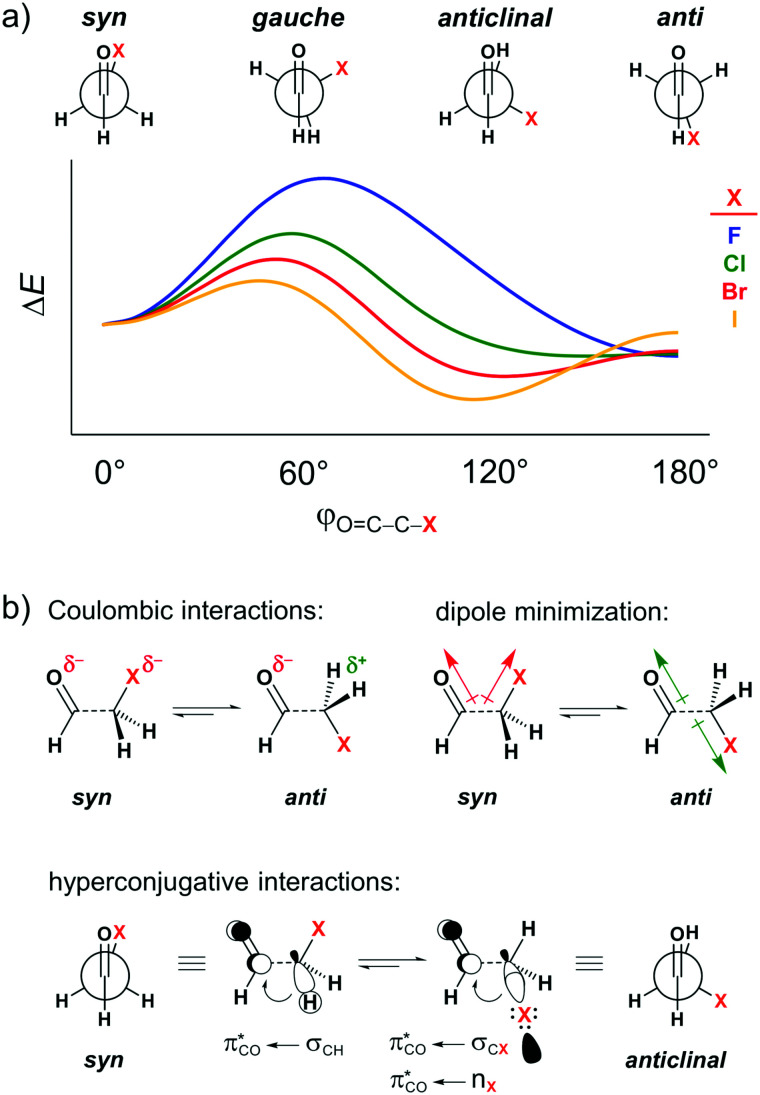
(a) Stationary points in the energy profile for rotation around the C–C bond of haloacetaldehydes and (b) intramolecular interactions used to rationalize the conformational preferences.

Several theoretical^7^ and experimental^8^ studies have explored the conformational landscape of haloacetaldehydes OHC–CH_2_X (X = F, Cl, Br, I) as archetypal model systems to investigate the main intramolecular interactions involving the carbonyl group. The rotation around the C–C bond in these systems results in the energy profile schematically illustrated in Fig. 1a. The relative conformational stability has typically been ascribed to dipolar interactions, that is, the *syn* conformer (*i.e.*, *φ*_O=C–C–X_ = 0°) experiences a strong electrostatic repulsion between the partially negatively charged oxygen and halogen atoms, which shifts the conformational equilibrium to *anti* (*i.e.*, *φ*_O=C–C–X_ = 180°; see Coulombic interactions in Fig. 1b).^7*a*^ This is equivalent to the explanation that the O=C and C–X bond dipoles in the O=C–C–X arrangement achieve the most or least unfavorable dipole–dipole interaction in the case of maximum (*syn*) or minimal (*anti*) overall dipole moment (see dipole minimization in Fig. 1b).^9^ The same rationalization has also been used to explain conformational energies in other systems containing polar groups, such as in the anomeric effect.^10^ Additionally, for heavier haloacetaldehydes, it is argued that the global energy minimum gradually shifts from the *anti* to the *anticlinal* conformation (*i.e.*, *φ*_O=C–C–X_ = 120°) because of the better donor ability of the σ_CX_ orbital to engage in stabilizing orbital interaction with the π*_CO_ orbital of the carbonyl group as X goes from F to I (see hyperconjugative interactions in Fig. 1b).^7*a*,11^

The above concept of dipolar repulsion depicts the electrostatic interaction as deriving from the sum of pairwise interactions between atoms that follows Coulomb's law for the respective point charges; this same representation can also be found in organic chemistry textbooks.^12^ However, the validity of such an oversimplified picture, the treatment of atoms as point charges, has been questioned in the literature. It can lead to incorrect predictions in, for instance, rationalizing the factors governing the conformational preferences of small organic molecules,^13^ chemical bonding,^14^ and non-covalent interactions.^15^ Molecules are characterized by a more complex charge distribution of nuclei and a 3-dimensional electron charge density that, more often than not, does not behave as a collection of point charges.^14*b*^ For example, some diatomic molecules, such as N_2_ and O_2_, would not be bound without the contribution from the attractive electrostatic component,^14*a*^ while the negative end of the dipole moment vector of the CO molecule lies on the partially positively charged carbon because the σ C–O HOMO has a large, lone-pair like lobe, colinear with, and pointing away from, the CO molecule.^16^

In this work, we have, therefore, analyzed the rotational isomerism of haloacetaldehydes OHC–CH_2_X (X = F, Cl, Br, and I, see Fig. 1) within the framework of quantitative Kohn–Sham molecular orbital (KS-MO) theory to reveal the physical mechanism behind the conformational preferences. In particular, we have investigated the role of dipolar repulsion as compared to other features in the bonding mechanism. Our bonding analyses show how the overall trend in the rotational energy profiles is set by the combined effects of Pauli repulsion, orbital interactions, and electrostatic interactions. Pauli repulsion, as will be explained, introduces a rotational barrier around *gauche* which separates minima at *syn* and *anti*. In addition, orbital interactions can pull the *anti* minimum towards a more *anticlinal* conformation to maximize hyperconjugation. And, only for X = F, not for X = Cl–I, electrostatic interactions push the preference from *syn* to *anti*. Our results furthermore reveal how this trend in electrostatic interactions is related to the compact nature of F, which shows behavior that is reminiscent of a partially negative point charge that has Coulomb repulsion with the partially negative O. At variance, the more diffuse nature of the heavier halogens X = Cl–I causes a more pronounced overlap of charge densities between O and X, which goes with a breakdown of the point-charge picture. The most striking consequence is a net stabilizing electrostatic interaction due to the reduced repulsion between the overlapping O and X densities in combination with significant stabilization of these densities by the nuclei of the other atom.

## Methods

### Computational details

All calculations were performed using the Amsterdam Density Functional (ADF) software package.^17^ The geometry of the stationary points and energy profile along rotation around the C–C bond of the haloacetaldehydes OHC–CH_2_X (X = F, Cl, Br, and I) were calculated using relativistic, dispersion-corrected density functional theory at ZORA-BP86-D3(BJ)/QZ4P. This approach comprises the generalized-gradient approximation of Becke (B) and Perdew (P86)^18^ augmented by Grimme's D3 dispersion correction^19^ using the damping function proposed by Becke and Johnson.^20^ Scalar relativistic effects are accounted for using the zeroth-order regular approximation (ZORA).^21^ Molecular orbitals (MO) were expanded in a large uncontracted set of Slater type orbitals (STOs) containing diffuse functions: QZ4P.^22^ The basis set is of quadruple-ζ quality augmented with polarization functions, *i.e.*, two 2p and two 3d sets on H, two 3d and two 4f sets on C, O, F; three 3d and two 4f sets on Cl, two 4d and three 4f sets on Br, one 5d and three 4f sets on I. All electrons were included in the variational process, *i.e.*, no frozen core approximation was applied. The accuracies of the fitting scheme (ZLM fit^23*a*^ for all computations except the decomposition of the electrostatic interaction term, as shown in eqn (3), which, for technical reasons, was performed with the STO fitting scheme^23*b*^) and the integration grid (Becke grid)^24^ were set to ‘VERY GOOD’. All optimized structures were confirmed to be true minima (no imaginary frequencies) or transition states (only one imaginary frequency) through vibrational analyses.^25^

The BP86-D3(BJ) functional has been previously shown to accurately reproduce the rotational profiles of 1,2-disubstituted ethanes.^26^ Herein, we have computed additional high-level relativistic CCSD(T) reference data (see Table S1 in the ESI†). Comparison with our ZORA-BP86-D3(BJ)/QZ4P results confirms that trends in homolytic C–C bond dissociation energies and, importantly, conformational preferences of haloacetaldehydes computed with our DFT approach agree well with those from the *ab initio* benchmark CCSD(T).

### Activation strain model and energy decomposition analysis

**Fig. 2:**
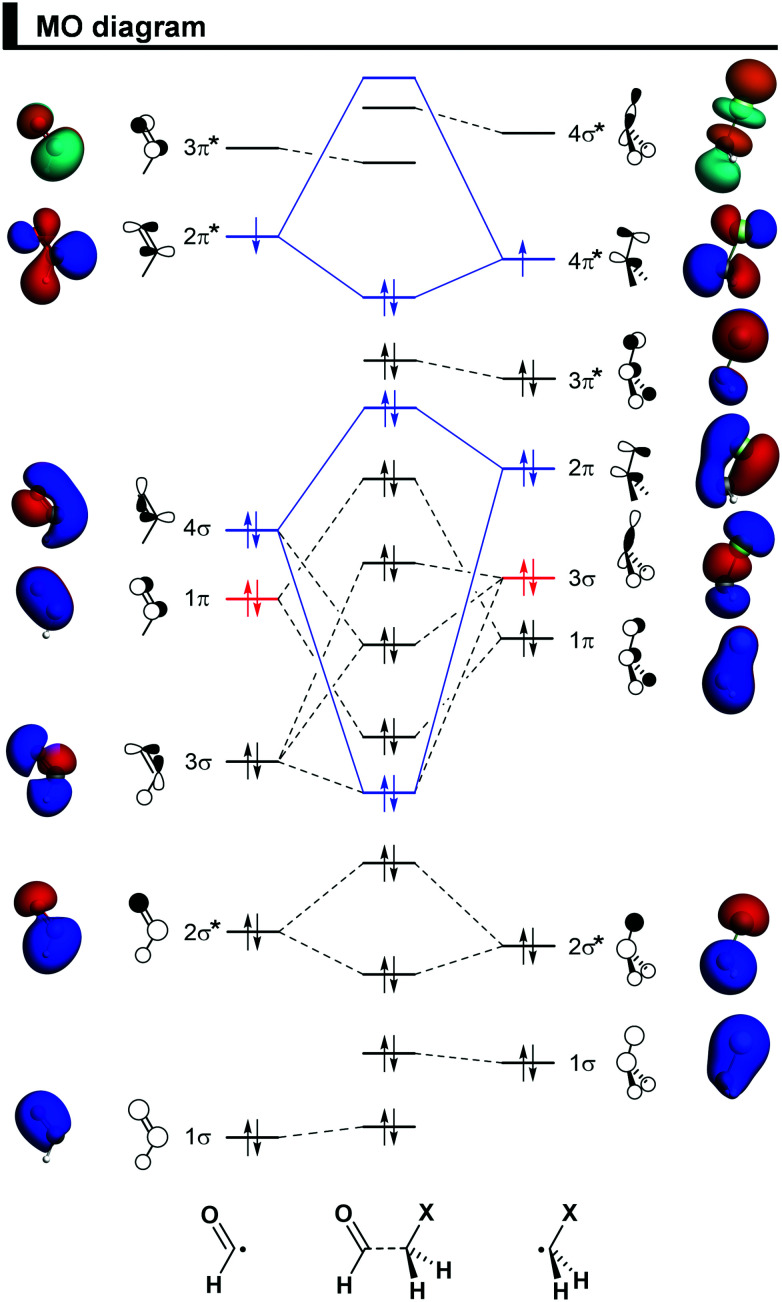
Schematic MO diagram for the formation of the *syn*-haloacetaldehydes OHC–CH_2_X (X = F, Cl, Br, I) from two open-shell fragments, OHC˙ and CH_2_X˙, along with the fragment molecular orbitals (FMO) depicted as quantitative 3D plots (isovalue = 0.04 a.u.) for the representative *syn*-chloroacetaldehyde, computed at ZORA-BP86-D3(BJ)/QZ4P. Note that the overlap between the closed-shell 1π OHC˙ and 3σ CH_2_X˙ orbitals (in red) builds up from *φ*_O=C–C–X_ = 0° to 90° and causes the central rotational barrier (see Fig. 4).

To understand how the OHC–CH_2_X bonding mechanism determines conformational preferences, we have analyzed this bond explicitly for all four haloacetaldehydes in terms of two open-shell fragments, OHC˙ and CH_2_X˙, forming a C–C electron-pair bond (see Fig. 2) in various conformations (*i.e.*, by varying the *φ*_O=C–C–X_ dihedral angle from 0° to 180°). The overall bond energy Δ*E* has been divided into two major components using the activation strain model (ASM),^27^ Δ*E*_strain_ and Δ*E*_int_, and projected these values onto *φ*_O=C–C–X_ [eqn (1)].

1 Δ*E* = Δ*E*_strain_ + Δ*E*_int_

In this equation, the strain energy Δ*E*_strain_ results from the distortion of the two open-shell fragments from their equilibrium structure to the geometry they acquire in the overall molecule, and Δ*E*_int_ is the actual interaction between the deformed fragments. The interaction energy Δ*E*_int_ was further decomposed using canonical energy decomposition analysis for open-shell fragments (EDA)^28^ into four physically meaningful energy terms [eqn (2)]:

2 Δ*E*_int_ = Δ*V*_elstat_ + Δ*E*_Pauli_ + Δ*E*_oi_ + Δ*E*_disp_

The Δ*V*_elstat_ term corresponds to classical electrostatic interaction between the unperturbed charge distributions of the (deformed) fragments and is usually attractive. The Δ*V*_elstat_ term can be further divided into four components [eqn (3)]:^28*a*^3
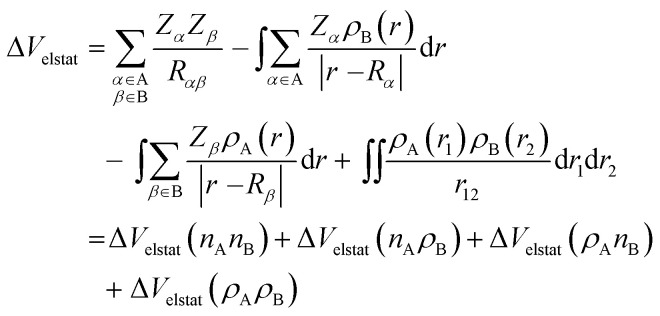
where A and B stand for OHC˙ and CH_2_X˙, respectively. The first term is the electrostatic repulsion between the nuclei of fragments A and B, Δ*V*_elstat_(*n*_A_*n*_B_); the second and third terms are the electrostatic attraction between the nuclei of fragment A and the electron density of fragment B, Δ*V*_elstat_(*n*_A_*ρ*_B_), and *vice versa*, Δ*V*_elstat_(*ρ*_A_*n*_B_); while the last term is the electrostatic repulsion between the electron densities of fragments A and B, Δ*V*_elstat_(*ρ*_A_*ρ*_B_).

The steric Pauli repulsion Δ*E*_Pauli_ comprises the destabilizing interaction between occupied closed-shell orbitals of both fragments due to the Pauli principle. The stabilizing orbital interactions Δ*E*_oi_ accounts for electron-pair bonding, charge transfer (*i.e.*, donor–acceptor interactions between occupied orbitals on one fragment and unoccupied orbitals on the other fragment), and polarization (*i.e.*, empty-occupied orbital mixing on one fragment due to the presence of the other fragment). The dispersion energy Δ*E*_disp_ corresponds to the dispersion corrections as introduced by Grimme *et al.*^19^

For the purpose of clarity, all above-mentioned energy terms along rotation around the C–C bond are considered relative to the *syn* conformation (*i.e.*, *φ*_O=C–C–X_ = 0°) and represented as a ΔΔ*E*. To facilitate the analyses, the ASM and EDA were performed using the PyFrag 2019 program.^29^

## Results and discussion

### Rotational energy profiles

**Fig. 3:**
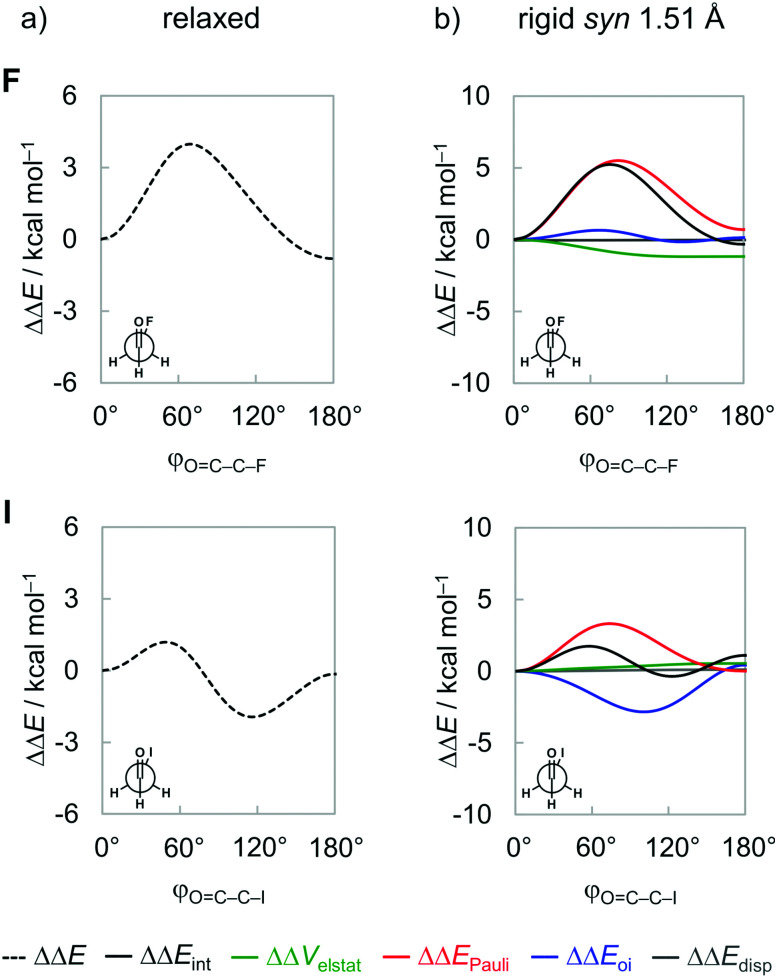
Rotational energy profile as a function of the *φ*_O=C–C–X_ dihedral angle of the fluoro and iodoacetaldehydes. (a) Fully relaxed rotation around the C–C bond, and (b) energy decomposition analysis (EDA) for rigid rotation in *syn* geometry but with a fixed C–C distance set to 1.51 Å (as in the conformer with the shortest C–C bond length, *i.e.*, the *syn*-iodoacetaldehyde). Energy terms relative to the *syn* conformer, ΔΔ*E*, computed at ZORA-BP86-D3(BJ)/QZ4P.

The energy profiles for half a rotation around the C–C bond, that is, from *φ*_O=C–C–X_ = 0° to 180°, of the fluoro and iodoacetaldehydes from our ZORA-BP86-D3(BJ)/QZ4P calculations are given in Fig. 3 (full data on all model systems can be found in Fig. S1 in the ESI†). Note that only half a rotation is shown because the energy profile from 180° to 360° mirrors the one from 0° to 180°. From Fig. 3a, we note the well-known energy profile of these systems which features two minimum-energy conformations: (i) one is always the *syn* conformer and (ii) the second one gradually changes its geometric character from *anti* to *anticlinal*, as X goes from F to I. This latter conformer is the global energy minimum in all cases, in line with earlier reports in the literature.^8^ Furthermore, the energy profile for half a rotation of the fluoroacetaldehyde has a onefold rotational barrier with one energy maximum around the *gauche* orientation (*i.e.*, *φ*_O=C–C–X_ = 70°), whereas the iodoacetaldehyde has a twofold rotational barrier with energy maxima at the *gauche* and *anti*-conformations.

To understand the origin of the conformational energy differences, the various contributors to the bond energy along internal rotation around the C–C bond were analyzed by applying the activation strain model (ASM)^27^ with canonical energy decomposition analysis (EDA,^28^ see Methods section). At this point, it is important to emphasize that the energy components are highly dependent on the geometry and the distance between the fragments.^27*a*^ In previous studies,^26^ we have shown that the interpretation of fully relaxed rotational energy profiles (*i.e.*, where all geometrical parameters are flexible to optimize during rotation) can be misleading because they comprise both, the change in the interaction terms due to the mutual reorientation of the two fragments along the internal rotation as well as the change in the interaction terms due to geometrical relaxation in response to the former changes. Along the relaxed rotation around the C–C bond of 1,2-dihaloethanes, the C–C distance expands or contracts as a result of the steric Pauli repulsion experienced in each conformation.^26^ When the C–C bond is shorter, the various orbital and electrostatic interactions are maximized, and the opposite occurs when the C–C bond is longer. Therefore, the changes manifested in the interaction energy components are just a consequence of the C–C bond distance variation. Similar behavior is observed in the relaxed rotation of the haloacetaldehydes studied herein (see Fig. S2, ESI†). Thus, to properly identify causalities in rotational profiles, it is necessary to perform the analysis at a rigid rotation around the C–C bond, that is, where all geometry parameters but the *φ*_O=C–C–X_ torsion angle are kept unchanged. To this end, we take the *syn* conformer of each haloacetaldehyde at its equilibrium geometry and rotate it with fixed C–C bond length and OHC˙ and CH_2_X˙ geometries to the *anti*-conformation. To compare all molecules on a more equal footing, the rotation of all haloacetaldehydes is performed from the same C–C distance set to 1.51 Å (as in the conformer with the shortest C–C bond length, *i.e.*, the *syn*-iodoacetaldehyde; see Fig. 3b). We note that, although physically plausible, our choices of geometrical constraints might seem somewhat arbitrary. We have, therefore, verified that all trends and conclusions that play a role in the following discussion are not affected if other plausible choices of fragment geometry and C–C distance are made. The same overall trend is found if the rigid rotation is performed from their equilibrium *syn* or *anti* geometries or in a longer C–C bond length (see Fig. S3, ESI†).

Inspection of the EDA plots in Fig. 3b reveals three major features about the rotational isomerism of haloacetaldehydes: (i) a barrier around *gauche* because of an increased steric Pauli repulsion ΔΔ*E*_Pauli_ (red curve) that separates *anti* and *syn* energy minima; (ii) the global energy minimum shifts from *anti* to *anticlinal* as X goes from F to I to maximize stabilizing orbital interactions ΔΔ*E*_oi_ (blue curve); and (iii) the only contribution from electrostatic interactions ΔΔ*V*_elstat_ (green curve) is to stabilize the *anti*-form for X = F. Note that the dispersion energy ΔΔ*E*_disp_ (grey curve) remains nearly constant upon rotation around the C–C bond and, therefore, does not contribute to the overall trend in the rotational energy profiles. We recall that all energy terms are represented as a ΔΔ*E* relative to the *syn* conformer. Thus, the strain energy Δ*E*_strain_ vanishes in this analysis because it is constant for geometrically frozen fragments; that is, ΔΔ*E*_strain_ is zero and ΔΔ*E*_int_ = ΔΔ*E*. The absolute energy values of the main conformations are provided in Table S2 in ESI.† In the following, we address each one of the abovementioned features and their underlying physical mechanism individually.

### Steric Pauli repulsion and rotational barriers

**Fig. 4:**
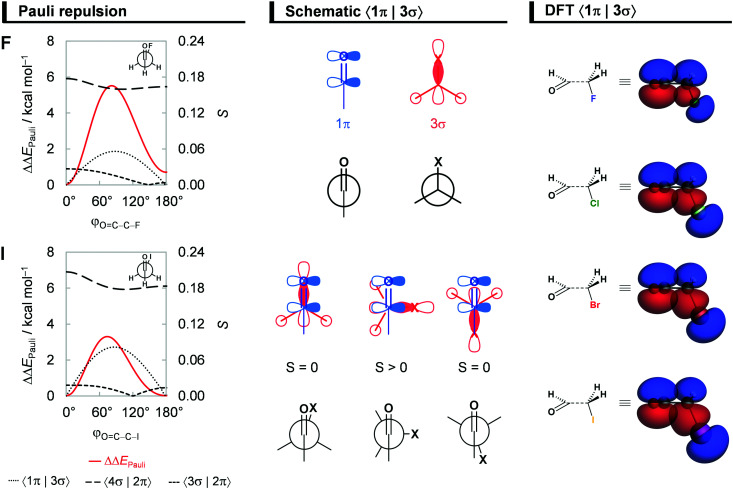
Steric Pauli repulsion ΔΔ*E*_Pauli_ and the most significant occupied–occupied orbital overlaps *S* as a function of the *φ*_O=C–C–X_ dihedral angle of the fluoro and iodoacetaldehydes, schematic representation of the 〈1π|3σ〉 overlap for *φ*_O=C–C–X_ = 0° (*syn*), 90°, and 180° (*anti*), and the 〈1π|3σ〉 overlap depicted as 3D plots (isosurface at 0.03 a.u.) for all haloacetaldehydes. Analysis in rigid rotation in *syn* geometry but with C–C bond distance set to 1.51 Å. Energy terms relative to the *syn* conformer, ΔΔ*E*, computed at ZORA-BP86-D3(BJ)/QZ4P.

Our analysis of rotational energy profiles reveals that steric Pauli repulsion ΔΔ*E*_Pauli_ is the dominant term behind main rotational trends, namely, the central rotational barrier and the fact that both *anti* (for heavier halogens hyperconjugation pulls *anti* towards *anticlinal*, *vide infra*) and *syn* are energy minimum conformers. This is one more example that highlights the important role of steric Pauli repulsion in controlling rotational landscapes of organic molecules.^26,30^ As seen from Fig. 3b, ΔΔ*E*_Pauli_ is a minimum at the *syn* and *anti*-conformations and goes to a maximum when the OHC˙ and CH_2_X˙ fragments are nearly perpendicular (*i.e.*, *φ*_O=C–C–X_ is *ca.* 70–80°). The most significant closed-shell–closed-shell overlap contributing to the trend in ΔΔ*E*_Pauli_ arises between the C=O π-bonding OHC˙ FMO with the C–X σ-bonding CH_2_X˙ FMO, that is, 〈1π|3σ〉. The MO diagram with the valence orbitals of the OHC˙ and CH_2_X˙ fragments is provided in Fig. 2 and the 〈1π|3σ〉 overlap is illustrated in more detail in Fig. 4. Note that 〈1π|3σ〉 is zero at the *syn* and *anti*-conformations as the 3σ orbital of CH_2_X˙ overlaps symmetrically with the nodal plane region of the 1π orbital of OHC˙ (see Fig. 4; see also Fig. S4 for the orbital overlap depicted as 3D plots, ESI†). But, as the dihedral angle *φ*_O=C–C–X_ is rotated in between, 〈1π|3σ〉 deviates from zero and achieves a maximum around 90° (see Fig. 4). This maximum becomes larger and the effect is more pronounced as the 3σ orbital becomes more diffuse, *i.e.*, for heavier halogens X. For example, at *φ*_O=C–C–X_ = 90°, 〈1π|3σ〉 varies from a value of 0.06 to 0.08 along X = F to I (see Fig. 4). Similar behavior is found for the 〈1π|2π〉 and 〈3σ|1π〉 overlap integrals (see Fig. S5, ESI†).

It is interesting to observe that ΔΔ*E*_Pauli_ along rotation around the C–C bond favors the *syn* conformer, where the most electron-rich atoms on each fragment are in closest proximity. The reason for this is twofold. Firstly, the overlap between FMOs that possess O and X lone-pair character, 〈3σ|2π〉 and 〈4σ|2π〉, expected to give a more destabilizing ΔΔ*E*_Pauli_ at the *syn*, only slightly changes upon rotation around the C–C bond (see Fig. 4). This is because the overlapping region is largest in between the two carbons, on the C–C bond region, and, therefore, varies less as a function of the *φ*_O=C–C–X_ torsional angle (see Fig. S4 for the 3D plots of 〈3σ|2π〉 and 〈4σ|2π〉, ESI†). Secondly, the 3π* orbital, one of the main contributors to the Pauli repulsion in the rotational barrier of 1,2-dihaloethanes,^26^ is actually empty in the OHC˙ fragment (see Fig. 2) and, thus, 〈3π*|3π*〉 does not give rise to any Pauli repulsion between the OHC˙ and CH_2_X˙ fragments. Therefore, the trends in steric Pauli repulsion of haloacetaldehydes stem mostly from the 〈1π|3σ〉 overlap integral (Fig. 4), which is zero at the *syn* and *anti*-conformations.

### Hyperconjugative interactions

**Fig. 5:**
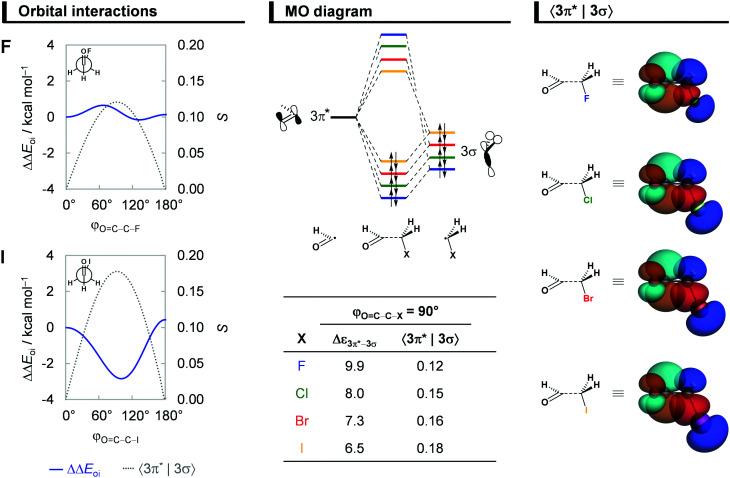
Orbital interactions ΔΔ*E*_oi_ as a function of the *φ*_O=C–C–X_ dihedral angle of the fluoro and iodoacetaldehydes, MO diagram along with the orbital energy gap (in eV) and overlap for the donor–acceptor interaction between the unoccupied 3π* orbital of OHC˙ and the occupied 3σ orbital of CH_2_X˙ (isosurface at 0.03 a.u.) of all haloacetaldehydes. Analysis in rigid rotation in *syn* geometry but with C–C distance set to 1.51 Å. Energy terms relative to the *syn* conformer, ΔΔ*E*, computed at ZORA-BP86-D3(BJ)/QZ4P.

Next, we comment on the role of orbital (hyperconjugative) interactions ΔΔ*E*_oi_ to the conformational energies. In agreement with previous reports in the literature,^7*a*,11^ the hyperconjugation becomes increasingly more stabilizing at the *anticlinal* conformer for heavier haloacetaldehydes (see Fig. 5). This is the reason why the global energy minimum conformation gradually shifts from *anti* to *anticlinal* as X goes from F to I. The stabilization due to ΔΔ*E*_oi_ results predominantly from a charge transfer into the empty C=O π*-antibonding OHC˙ FMO from the occupied C–X σ-bonding CH_2_X˙ FMO, that is, the 3π*–3σ interaction (see Fig. 5). Additional but less stabilizing contribution from the 3π*–2π interaction is given in Fig. S5 and Table S3 (ESI†). These orbital interactions are more stabilizing for the iodoacetaldehyde because of both smaller orbital energy gap and larger orbital overlap (*e.g.*, Δ*ε*_3π*–3σ_ = 9.9 and 6.5 eV, and 〈3π*|3σ〉 = 0.12 and 0.18 for X = F and I, respectively). As the electronegativity of the halogen atom decreases from F to I,^31^ the 3σ orbital becomes higher in energy, which leads to a smaller energy gap with the 3π* orbital of OHC˙. The associated 〈3π*|3σ〉 orbital overlap is largest at *φ*_O=C–C–X_ = 90° (Fig. 5; see Fig. S5 in ESI† for data along the rotation of the C–C bond) and increases as the 3σ orbital becomes more diffuse, due to the larger *n*p atomic orbital of heavier halogen atoms. These findings consolidate earlier studies on the role of hyperconjugative orbital interactions.^7*a*,11^

### Electrostatic interactions and dipolar repulsion

Finally, we address the small role of electrostatic interactions ΔΔ*V*_elstat_ to the rotational energy profiles, which, for X = F and I, favors *anti* and *syn*, respectively (see Fig. 3b). As will become clear in the following, this difference in preference originates from the small, compact nature of fluorine and the large, more diffuse nature of iodine. The small nucleus and compact electron density render the fluorine atom a point-charge-like behavior, which gradually fades as one goes down group 17 in the periodic table. The large nucleus and more diffuse electron density of the iodine atom result in more electrostatic attraction with the electron density and nucleus of the oxygen atom, respectively, as well as less repulsion between electron densities compared to point charges. This results in the rotational trends mentioned above, that is, along the ΔΔ*V*_elstat_ curve, the *anti* is preferred for X = F to reduce electrostatic repulsion, whereas the opposite is observed for X = I, the preference shifts to *syn* to enhance electrostatic attraction.

**Fig. 6:**
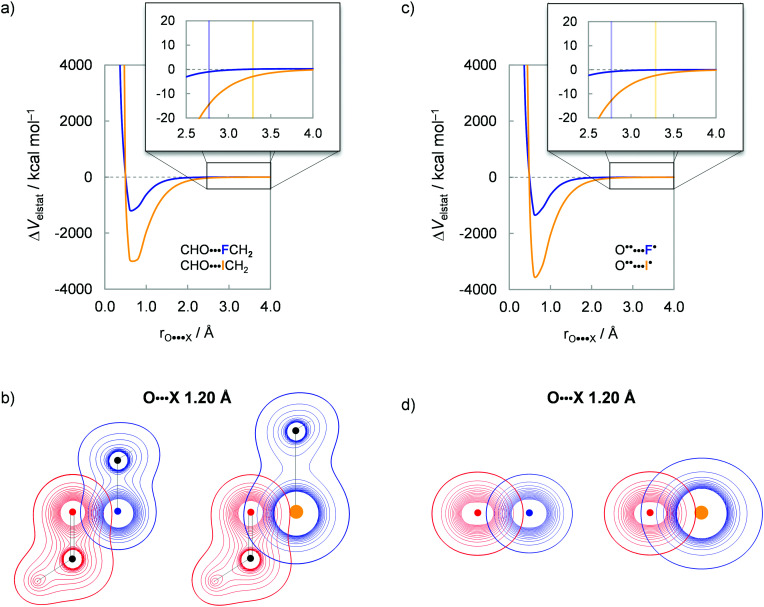
Electrostatic interactions Δ*V*_elstat_ between oxygen and halogen atoms. (a) Δ*V*_elstat_ as a function of the *r*_O⋯X_ distance and (b) density contours from −0.9 to 0.9 Bohr^−3^ for the lateral approach of the OHC˙ and CH_2_X˙ fragments. (c) Δ*V*_elstat_ as a function of the *r*_O⋯X_ distance and (d) density contours from −0.9 to 0.9 Bohr^−3^ for the approach of the bare O˙˙ and X˙ atoms in their triplet and doublet valence configuration, respectively. Vertical lines indicate the *r*_O⋯X_ separation in the geometry of the corresponding haloacetaldehyde. Computed at ZORA-BP86-D3(BJ)/QZ4P.

To specifically evaluate the magnitude of the electrostatic interaction between the oxygen and halogen atoms, we approach the OHC˙ and CH_2_X˙ fragments not by forming a C–C electron-pair bond, but along the O⋯X distance (where X = F and I), as shown in Fig. 6a and b. The Δ*V*_elstat_ energy becomes repulsive only at a very short internuclear distance (*r*_O⋯X_ < 0.8 Å), which stems mostly from the nuclei–nuclei repulsion.^14*b*^ Note that the physical nature of the charge densities is different from point charges, and the former yields less electrostatic repulsion than the latter.^14*a*^ Furthermore, Δ*V*_elstat_ is less stabilizing for X = F than X = I all along the O⋯X distance, even at their *r*_O⋯X_ distance in the corresponding haloacetaldehydes (see vertical lines in Fig. 6a). The same overall trend is observed if one analyzes the electrostatic interaction by approaching the OHC˙ and CH_2_X˙ fragments along the C=O⋯X–C bond axis (see Fig. S6, ESI†). This can be traced back to the electrostatic interaction between the bare O˙˙ and X˙ atoms in their triplet and doublet valence configuration, respectively (see Fig. S7, ESI†). The overlap between electron densities and nuclei of the OHC˙ and CH_2_X˙ fragments is better for X = I because the electron density of the iodine atom is more diffuse and its nucleus is larger than the fluorine atom (Fig. 6d), resulting in stronger electrostatic attraction with the oxygen atom (Fig. 6c). Therefore, the electrostatic interaction Δ*V*_elstat_ between OHC˙ and CH_2_X˙ is predominantly attractive and increases in magnitude from X = F to I, leading to a breakdown of the point-charge picture.

However, at short distances, even the compact fluorine atom can deviate from point-charge behavior. Along the series of haloacetaldehydes, this can be achieved by artificially shortening the C–X bond length and, therefore, the O⋯X separation. Table 1 shows the ΔΔ*V*_elstat_ energy of the *syn* relative to the *anti*-conformation. We recall that Δ*V*_elstat_ is predominantly attractive and, therefore, positive (*i.e.*, less stabilizing) values of ΔΔ*V*_elstat_ indicate that *syn* is electrostatically less favorable than *anti*, whereas negative (*i.e.*, more stabilizing) values of ΔΔ*V*_elstat_ denote the opposite. By decreasing the O⋯X distance, the overlap between electron densities and nuclei of OHC˙ and CH_2_X˙ becomes more effective, resulting in more stabilizing electrostatics at the *syn* conformer for all haloacetaldehydes (see Table 1, column “C–X –0.3 Å”). This is further corroborated by the fact that the *anti* preference dominates with the elongation of the C–X bond length and, therefore, of the O⋯X distance (see Table 1, column “C–X +0.3 Å”). At a longer O⋯X separation, Δ*V*_elstat_ becomes less stabilizing, which shifts the electrostatic preference to *anti*. Thus, the electrostatic preference for *syn* or *anti* depends on the balance between the attractive and repulsive components of Δ*V*_elstat_ (see eqn (3) and Fig. S5 in ESI†). For X = I, Δ*V*_elstat_ is strongly stabilizing and prefers to be *syn* to maximize the overlap between the electron density on one with the nuclei on the other of the OHC˙ and CH_2_X˙ fragments. For X = F, on the other hand, the electrostatic attraction is weaker than for X = I and does not compensate for the electrostatic repulsion between nuclei and between densities on either fragment at *syn*, therefore, shifting the preference to *anti* (see Tables S2 and S4 for absolute and relative values, respectively, ESI†). The oversimplified concept of dipolar repulsion does not capture this interplay of electrostatic interactions. At variance with this rationale, the point-charge-like behavior of the fluorine atom along the ΔΔ*V*_elstat_ curve is *not* due to a larger electrostatic repulsion, but instead stems from the *attractive* electrostatic components that are less stabilizing than for larger halogens.

**Table 1:** *Syn* relative to *anti* electrostatic interactions ΔΔ*V*_elstat_ (in kcal mol^−1^) of haloacetaldehydes OHC–CH_2_X (X = F, Cl, Br, and I)^a^

X	C–X –0.3 Å	C–X equil^b^	C–X +0.3 Å
	ΔΔ*V*_elstat_	ΔΔ*V*_elstat_	ΔΔ*V*_elstat_
F	−0.4	1.2	2.4
Cl	−0.3	0.5	1.5
Br	−0.9	0.1	1.1
I	−1.7	−0.5	0.5

a Computed at ZORA-BP86-D3(BJ)/QZ4P.

b Data from the rigid rotation in *syn* geometry but with C–C distance set to 1.51 Å.

## Conclusions

The overall rotational energy profile of haloacetaldehydes OHC–CH_2_X (X = F, Cl, Br, and I) is set by an interplay of steric Pauli repulsion, which causes a barrier separating *syn*- and *anti*-minima, orbital interactions, which pull the *anti*-minimum towards *anticlinal* in order to optimize hyperconjugative overlap, and electrostatic interactions, which drive the preference to *anti* only in the case of X = F. This follows from our detailed analyses based on relativistic dispersion-corrected density functional theory at ZORA-BP86-D3(BJ)/QZ4P.

The results of our quantitative Kohn–Sham molecular orbital theory analyses reveal that steric Pauli repulsion is the causal term giving rise to the central rotation barrier between *syn*- and *anti*-minima, which reaches a maximum when the C=O and C–X bonds are nearly perpendicular, due to the maximum overlap in the four–electron interaction between the filled π orbital of the carbonyl group with the filled σ orbital of the halogenated methyl fragment. This closed-shell–closed-shell orbital overlap is zero at the *syn* and *anti*-conformations because the CH_2_X˙ σ orbital overlaps symmetrically with the nodal plane region of the OHC˙ π orbital. Therefore, Pauli repulsion is also the reason why the *syn* is an energy minimum conformer for all haloacetaldehydes. The global energy minimum, on the other hand, gradually shifts from *anti* to *anticlinal* as X goes from F to I because of the more stabilizing orbital interactions in the latter, in line with the currently accepted rationale. As the halogen atom increases in size, the orbital overlap becomes larger, and the orbital energy gap becomes smaller for the charge transfer to the empty π* orbital of the carbonyl group from the filled σ orbital of the halogenated methyl.

Electrostatic interactions are intuitively seen as the repulsion or attraction between atoms with partial charges of the same or opposite signs, respectively. As such, they are often represented as the sum of pairwise interactions that follows Coulomb's law for the respective point charges, which is the center of the concept of dipolar repulsion. We show that this oversimplified view is, however, only valid for compact atoms, in our model systems, the second-row fluorine atom. As the halogen atom increases in size and becomes more diffuse, it deviates more from the behavior of point charge to an atomic charge distribution. This results in a net stabilizing electrostatic interaction between O and X arising from both less repulsion between overlapping densities and more attraction between overlapping density and nucleus. Therefore, while the electrostatic energy favors *anti* for X = F to reduce O(*ρ*^−^)⋯(*ρ*^−^)F and O(⊕)⋯(⊕)F repulsions, it favors *syn* for X = I to enhance O(⊕)⋯(*ρ*^−^)I and O(*ρ*^−^)⋯(⊕)I attractions.

## Conflicts of interest

There are no conflicts to declare.

## Supplementary Material

CP-023-D1CP02502C-s001
