# Supplementary material for: Dipolar repulsion in α-halocarbonyl compounds revisited
Source: Phys Chem Chem Phys. 2021 Sep 1;23(37):20883–91. doi: 10.1039/d1cp02502c (PMC8479779; doi:10.1039/d1cp02502c)
Supplement: CP-023-D1CP02502C-s001 [file CP-023-D1CP02502C-s001.pdf]

Electronic Supplementary Material (ESI) for Physical Chemistry Chemical Physics.
This journal is © the Owner Societies 2021

Electronic Supplementary Information for:

Dipolar Repulsion in α-Halocarbonyl Compounds Revisited

Daniela Rodrigues Silva,[a,b] Lucas de Azevedo Santos,[a,b] Trevor A. Hamlin,[a] F. Matthias Bickelhaupt,*[a,c] Matheus P. Freitas,*[b] and Célia Fonseca Guerra*[a,d]

[a] Department of Theoretical Chemistry, Amsterdam Institute of Molecular and Life Sciences (AIMMS), Amsterdam Center for Multiscale Modeling (ACMM), Vrije Universiteit Amsterdam, De Boelelaan 1083, 1081 HV Amsterdam (The Netherlands)
E-mail: f.m.bickelhaupt@vu.nl, c.fonsecaguerra@vu.nl

[b] Departamento de Química, Instituto de Ciências Naturais Universidade Federal de Lavras, 37200-900, Lavras–MG (Brazil)
E-mail: matheus@ufla.br

[c] Institute for Molecules and Materials (IMM), Radboud University, Heyendaalseweg 135, 6525 AJ Nijmegen (The Netherlands)

[d] Leiden Institute of Chemistry, Gorlaeus Laboratories, Leiden University, Einsteinweg 55, 2333 CC Leiden (The Netherlands)

Table of contents

Table S1. Homolytic bond dissociation energies (in kcal mol^{-1}) for the reaction OHC–CH_2X → OHC$^•$ + $^•CH_2X$ (X = F, Cl, Br, and I).

Figure S1. Rotational energy profile as a function of the $\varphi_{O=C–C–X}$ dihedral angle of haloacetaldehydes OHC–CH_2X (X = F, Cl, Br, and I). a) fully relaxed rotation around the C–C bond, and b) energy decomposition analysis (EDA) for rigid rotation in *syn* geometry but with C–C distance set to 1.51 Å. Energy terms relative to the *syn* conformer, ΔΔ*E*, computed at ZORA-BP86-D3(BJ)/QZ4P.

Figure S2. a) Activation strain (ASA) and b) energy decomposition analyses (EDA) of the fully relaxed rotation around the C–C bond along with c) key bond length variations as a function of the $\varphi_{O=C–C–X}$ dihedral angle of haloacetaldehydes OHC–CH_2X (X = F, Cl, Br, and I). Energy terms and distances relative to the *syn* conformer, ΔΔ*E* and Δr, computed at ZORA-BP86-D3(BJ)/QZ4P.

Figure S3. Energy decomposition analysis (EDA) for rigid rotation around the C–C bond as a function of the $\varphi_{O=C-C-X}$ dihedral angle of haloacetaldehydes OHC–CH_2X (X = F, Cl, Br, and I). a) rigid rotation in *syn* geometry, b) rigid rotation in *syn* geometry but with C–C distance set to 1.51 Å (as in the *syn*-iodoacetaldehyde), c) rigid rotation in *anti*-geometry, and d) rigid rotation in *anti*-geometry but with C–C distance set to 1.52 Å (as in the *anti*-fluoroacetaldehyde). Energy terms relative to the *syn* conformer, $\Delta\Delta E$, computed at ZORA-BP86-D3(BJ)/QZ4P.

Table S2. EDA terms (in kcal mol^{-1}) of the main conformations of haloacetaldehydes OHC–CH_2X (X = F, Cl, Br, and I) in the rigid rotation around the C–C bond in *syn* geometry but with C–C distance set to 1.51 Å.

Figure S4. Key closed-shell–closed-shell overlaps between fragment molecular orbitals (FMOs, isosurface at 0.03 a.u.) in the *syn* and *anti*-conformers depicted as quantitative 3D plots for the chloroacetaldehyde analogue. Analysis in rigid rotation in *syn* geometry but with C–C bond distance set to 1.51 Å, computed at ZORA-BP86-D3(BJ)/QZ4P.

Figure S5. a) Electrostatic interaction energy components, b) Pauli repulsion and key occupied–occupied orbital overlaps, and c) orbital interactions and key unoccupied–occupied orbital overlaps as a function of the $\varphi_{O=C-C-X}$ dihedral angle of haloacetaldehydes OHC–CH_2X (X = F, Cl, Br, and I). Analysis in rigid rotation in *syn* geometry but with C–C distance set to 1.51 Å. Energy terms relative to the *syn* conformer, $\Delta\Delta E$, computed at ZORA-BP86-D3(BJ)/QZ4P.

Table S3. Orbital energy gap (in eV) and overlap of key unoccupied–occupied orbital interactions of haloacetaldehydes OHC–CH_2X (X = F, Cl, Br, and I).

Figure S6. Electrostatic interaction between oxygen and halogen atoms for the C=O•••X–C colinear approach of the $OHC^\bullet$ and $CH_2X^\bullet$ fragments. a) ΔV_{elstat} as a function of the $r_{O\bullet\bullet\bullet X}$ distance (vertical lines indicate $r_{O\bullet\bullet\bullet X}$ separation in the corresponding haloacetaldehyde); b) density contours from –0.9 to 0.9 $Bohr^{-3}$ for $OHC^\bullet$ and $CH_2X^\bullet$ fragments (X = F, I for upper and lower, resp.). Computed at ZORA-BP86-D3(BJ)/QZ4P.

Figure S7. Schematic diagram of the valence configuration of the $O^{\bullet\bullet}$ and $X^{\bullet}$ (X = F and I) atoms used for the electrostatic interaction analysis shown in Figure 6c,d in the main text, in which the bare atoms approach each other along the z axis. Note that this reflects the situation for the interaction between the full $OHC^{\bullet}$ and $CH_2X^{\bullet}$ fragments, the parts of which that have been cut away, here, in the $O^{\bullet\bullet}$ and $X^{\bullet}$ systems, are shown in faded grey.

Table S4. *Syn* relative to *anti* electrostatic interaction $\Delta\Delta V_{elstat}$ energy components (in kcal mol^{-1}) of haloacetaldehydes OHC–CH_2X (X = F, Cl, Br, and I).

Table S5. Cartesian coordinates (Å), energies (kcal mol^{-1}), and the number of imaginary vibrational frequencies (N_{imag}) of the stationary points in the energy profiles for rotation around the C–C bond of haloacetaldehydes OHC–CH_2X and the open-shell $HOC^{\bullet}$ and $CH_2X^{\bullet}$ fragments (X = F, Cl, Br, and I), computed at ZORA-BP86-D3(BJ)/QZ4P.

Table S1. Homolytic bond dissociation energies (in kcal mol^{-1}) for the reaction OHC–CH_2X → $OHC^{\bullet}$ + $^{\bullet}CH_2X$ (X = F, Cl, Br, and I).

X	CCSD(T)[a]		BP86-D3(BJ)[b]	
	syn	*anti(clinal)*[c]	*syn*	*anti(clinal)*[c]
**F**	85.4	86.7	81.6	82.5
**Cl**	83.2	84.3	79.5	80.4
**Br**	84.3	85.8	81.2	82.6
**I**	86.0	88.0	81.8	83.8

[a] Computed at ZORA-CCSD(T)/ma-def2-QZVPP[1]//ZORA-BP86-D3(BJ)/QZ4P, quasi-restricted orbitals (QROs) were used for open-shell fragments; computations at *ab initio* level were carried out using ORCA[2]. [b] Computed at ZORA-BP86-D3(BJ)/QZ4P. [c] The *anti*-conformer is the global energy minimum conformation for X = F, whereas the *anticlinal* conformation is the lowest in energy for X = Cl, Br, and I.

1 (a) E. van Lenthe, R. van Leeuwen, E. J. Baerends and J. G. Snijders, *Int. J. Quantum Chem.*, 1996, **57**, 281; (b) E. van Lenthe, E. J. Baerends and J. G. Snijders, *J. Chem. Phys.*, 1994, **101**, 9783; (c) K. Raghavachari, G. W. Trucks, J. A. People and M. Head-Gordon, *Chem. Phys. Lett.*, 1989, **157**, 479; (d) D. A. Pantazis, X. Chen, C. R. Landis and F. Neese, *J. Chem. Theory*, 2008, **4**, 908. (e) J. Zheng, X. Xu and D. G. Truhlar, *Theor. Chem. Acc.*, 2011, **128**, 295.

2 F. Neese, *WIREs Comput. Mol. Sci.*, 2012, **2**, 73.

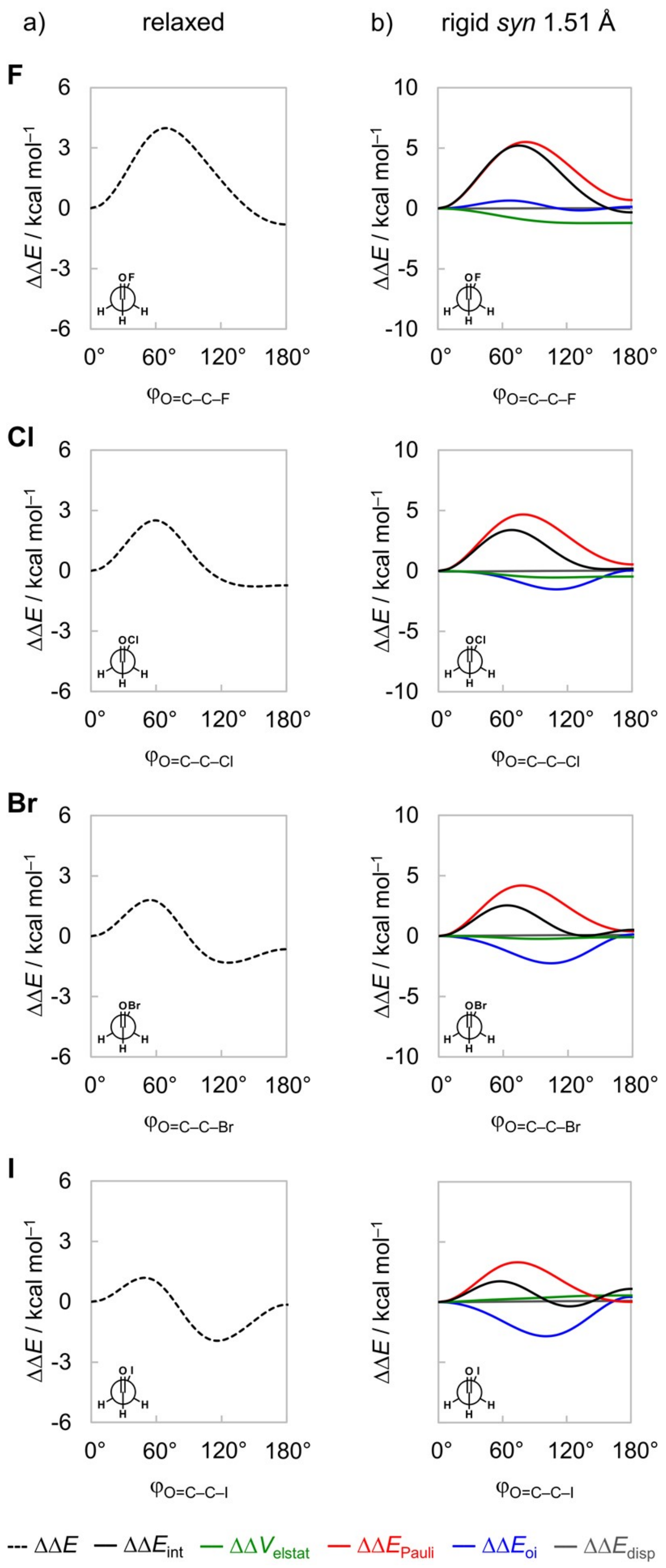

Figure S1. Rotational energy profile as a function of the $\varphi_{O=C–C–X}$ dihedral angle of haloacetaldehydes OHC–CH_2X (X = F, Cl, Br, and I). a) fully relaxed rotation around the C–C bond, and b) energy decomposition analysis (EDA) for rigid rotation in *syn* geometry but with C–C distance set to 1.51 Å. Energy terms relative to the *syn* conformer, $\Delta\Delta E$, computed at ZORA-BP86-D3(BJ)/QZ4P.

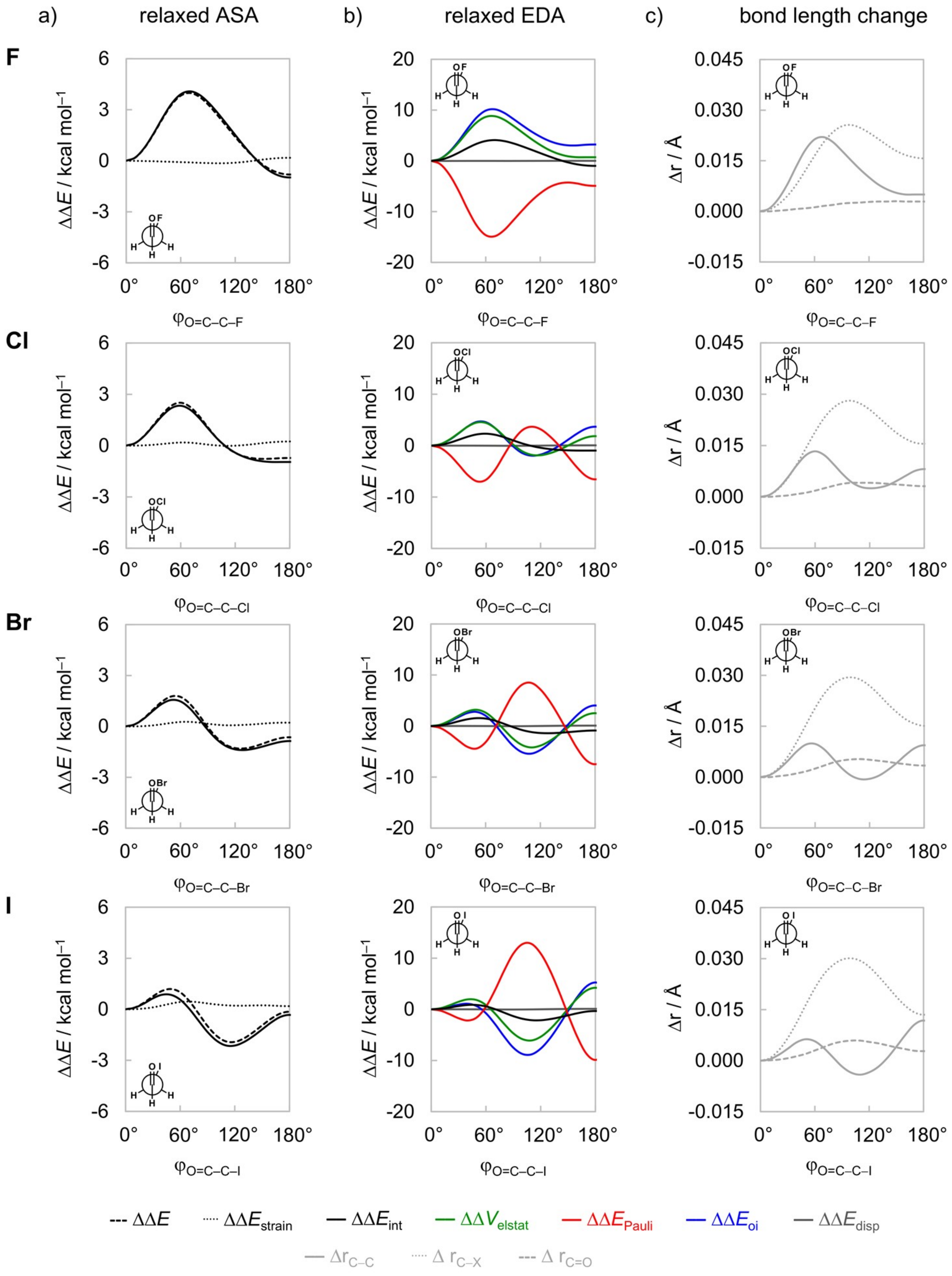

Figure S2. a) Activation strain (ASA) and b) energy decomposition analyses (EDA) of the fully relaxed rotation around the C–C bond along with c) key bond length variations as a function of the $\varphi_{O=C-C-X}$ dihedral angle of haloacetaldehydes OHC–CH_2X (X = F, Cl, Br, and I). Energy terms and distances relative to the *syn* conformer, $\Delta\Delta E$ and Δr, computed at ZORA-BP86-D3(BJ)/QZ4P.

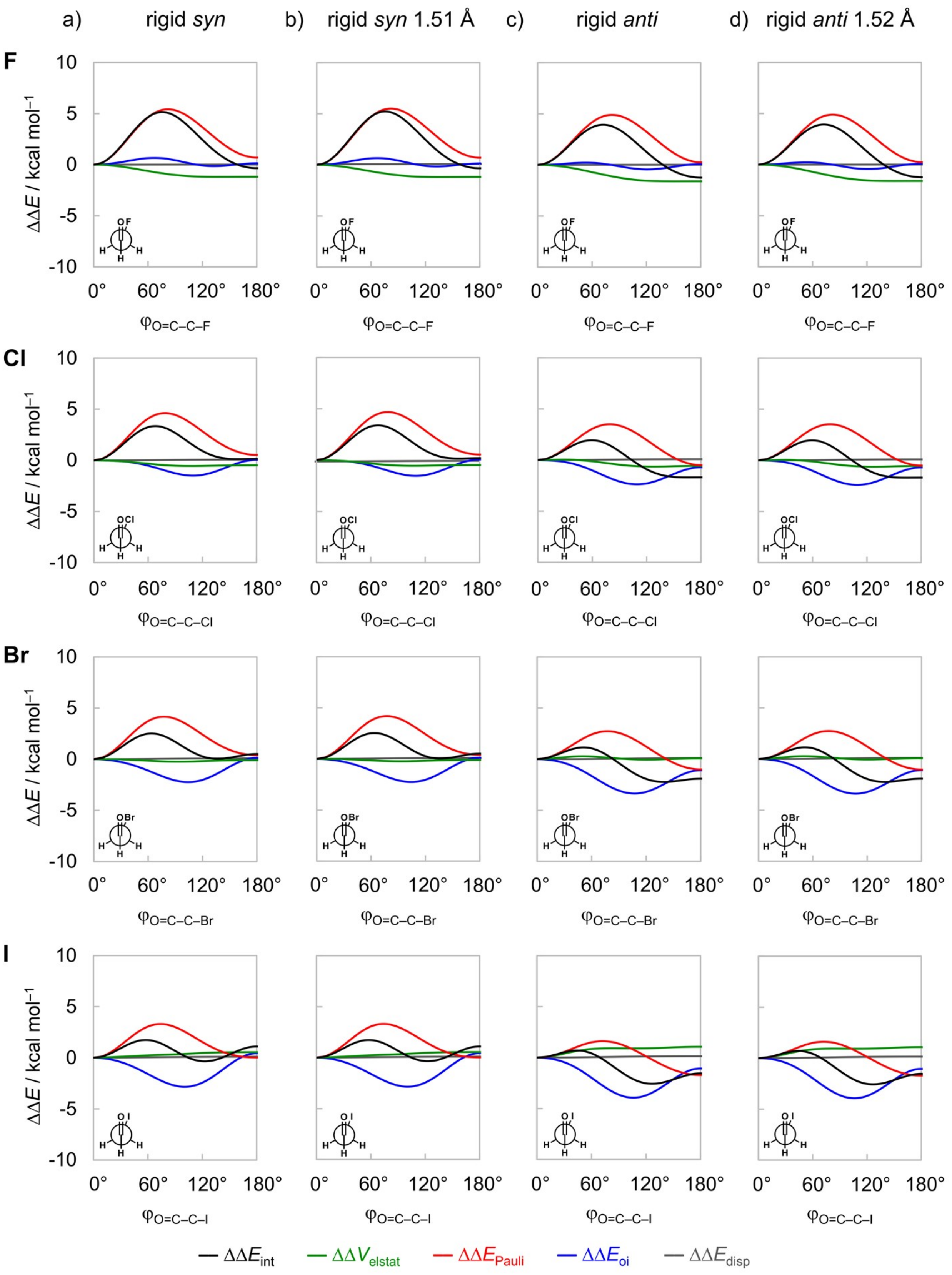

Figure S3. Energy decomposition analysis (EDA) for rigid rotation around the C–C bond as a function of the $\varphi_{O=C–C–X}$ dihedral angle of haloacetaldehydes OHC–CH_2X (X = F, Cl, Br, and I). a) rigid rotation in *syn* geometry, b) rigid rotation in *syn* geometry but with C–C distance set to 1.51 Å (as in the *syn*-iodoacetaldehyde), c) rigid rotation in *anti*-geometry, and d) rigid rotation in *anti*-geometry but with C–C distance set to 1.52 Å (as in the *anti*-fluoroacetaldehyde). Energy terms relative to the *syn* conformer, $\Delta\Delta E$, computed at ZORA-BP86-D3(BJ)/QZ4P.

Table S2. EDA terms (in kcal mol^{-1}) of the main conformations of haloacetaldehydes OHC–CH_2X (X = F, Cl, Br, and I) in the rigid rotation around the C–C bond in *syn* geometry but with C–C distance set to 1.51 Å.[a]

X	φ[b]	ΔE_{int}	ΔV_{elstat}	$\Delta V_{elstat}(n_A n_B)$[c]	$\Delta V_{elstat}(\rho_A n_B)$[c]	$\Delta V_{elstat}(n_A \rho_B)$[c]	$\Delta V_{elstat}(\rho_A \rho_B)$[c]	f[d]	ΔE_{Pauli}	ΔE_{oi}	ΔE_{disp}
F	**0**	–90.8	–181.1	36,222.3	–36,360.9	–36,304.7	36,263.2	–0.9	324.5	–232.5	–1.6
	**60**	–86.1	–181.8	35,711.7	–35,832.6	–35,791.5	35,731.6	–0.8	329.2	–231.9	–1.6
	**120**	–88.4	–182.3	35,032.5	–35,125.6	–35,102.8	35,014.5	–0.8	328.2	–232.6	–1.6
	**180**	–91.2	–182.3	34,813.2	–34,892.5	–34,874.5	34,772.7	–1.1	325.2	–232.4	–1.6
Cl	**0**	–90.5	–179.6	47,864.1	–48,044.2	–47,968.1	47,970.0	–1.2	327.9	–236.5	–2.3
	**60**	–87.2	–179.9	46,897.5	–47,041.3	–46,997.6	46,962.9	–1.3	332.1	–237.0	–2.3
	**120**	–89.6	–180.2	45,618.0	–45,704.6	–45,707.3	45,615.0	–1.0	330.8	–238.0	–2.3
	**180**	–90.3	–180.1	45,218.7	–45,276.8	–45,300.9	45,180.2	–1.2	328.5	–236.4	–2.3
Br	**0**	–91.7	–180.4	75,667.9	–75,955.0	–75,793.8	75,901.8	–1.2	330.3	–239.0	–2.6
	**60**	–89.2	–180.6	73,665.4	–73,875.7	–73,787.0	73,818.2	–1.4	334.0	–240.1	–2.5
	**120**	_91.4	–180.6	71,006.3	–71,095.1	–71,114.5	71,024.2	–1.3	332.7	–241.0	–2.5
	**180**	–91.2	–180.5	70,183.5	–70,212.0	–70,283.3	70,132.9	–1.5	330.7	–238.8	–2.5
I	**0**	–92.4	–181.8	100,432.6	–100,805.7	–100,584.4	100,777.3	–1.4	335.1	–242.8	–2.9
	**60**	–90.7	–181.5	97,469.6	–97,727.4	–97,618.0	97,696.0	–1.6	338.1	–244.4	–2.8
	**120**	–92.7	–181.3	93,535.1	–93,612.4	–93,672.2	93,569.6	–1.3	336.6	–245.3	–2.8
	**180**	–91.3	–181.2	92,318.6	–92,306.9	–92,448.7	92,257.3	–1.4	335.1	–242.4	–2.8

[a] Computed at ZORA-BP86-D3(BJ)/QZ4P. [b] The $\varphi_{O=C–C–X}$ dihedral angle (where X = F, Cl, Br, and I). [c] Sub index A for the OHC$^{\bullet}$ fragment and B for the $CH_2X^{\bullet}$ fragment. [d] Fitting for incompleteness of the ΔV_{elstat} term due to the use of density fitting.

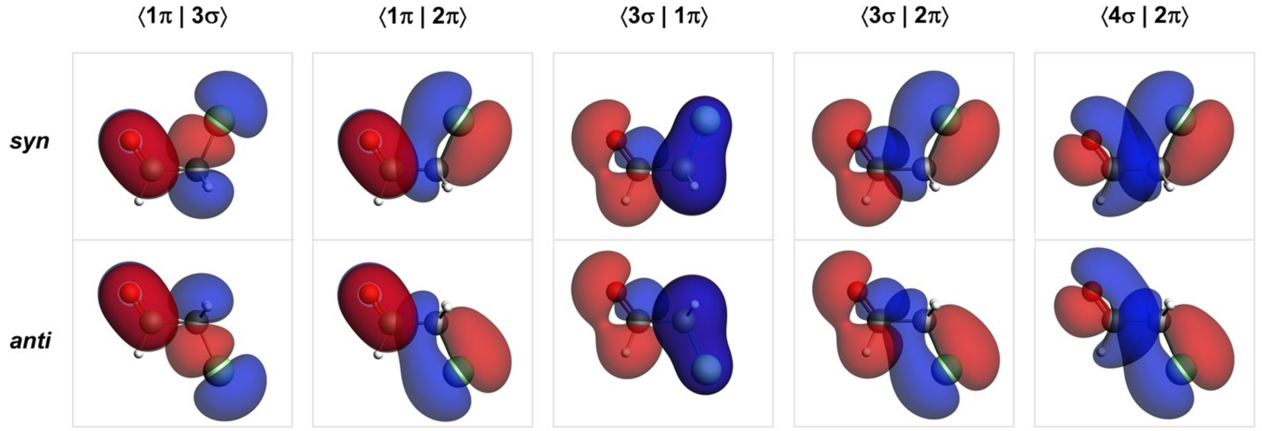

Figure S4. Key closed-shell–closed-shell overlaps between fragment molecular orbitals (FMOs, isosurface at 0.03 a.u.) in the *syn* and *anti*-conformers depicted as quantitative 3D plots for the chloroacetaldehyde analogue. Analysis in rigid rotation in *syn* geometry but with C–C bond distance set to 1.51 Å, computed at ZORA-BP86-D3(BJ)/QZ4P.

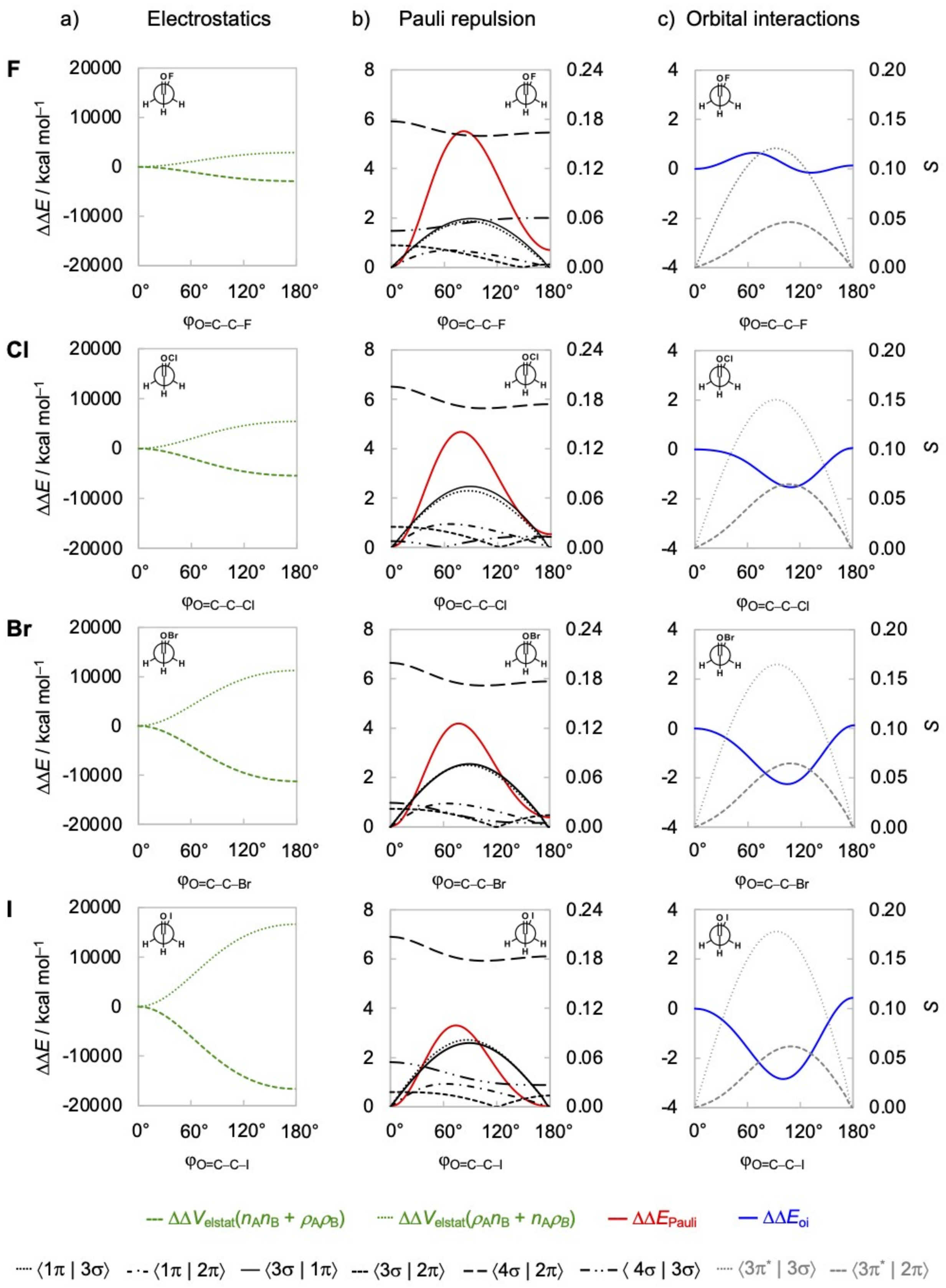

Figure S5. a) Electrostatic interaction energy components, b) Pauli repulsion and key occupied–occupied orbital overlaps, and c) orbital interactions and key unoccupied–occupied orbital overlaps as a function of the $\varphi_{O=C-C-X}$ dihedral angle of haloacetaldehydes OHC–CH_2X (X = F, Cl, Br, and I). Analysis in rigid rotation in *syn* geometry but with C–C distance set to 1.51 Å. Energy terms relative to the *syn* conformer, $\Delta\Delta E$, computed at ZORA-BP86-D3(BJ)/QZ4P.

Table S3. Orbital energy gap (in eV) and overlap of key unoccupied–occupied orbital interactions of haloacetaldehydes OHC–CH_2X (X = F, Cl, Br, and I).[a]

	$\varphi_{O=C-C-X}$ = 90° [b]			$\varphi_{O=C-C-X}$ = 100° [b]		
**X**	$\Delta\varepsilon_{3\pi^*-3\sigma}$ [c]	$\langle 3\pi^* \mid 3\sigma \rangle$ [c]	$S^2/\Delta\varepsilon$ x 10^3	$\Delta\varepsilon_{3\pi^*-2\pi}$ [c]	$\langle 3\pi^* \mid 2\pi \rangle$ [c]	$S^2/\Delta\varepsilon$ x 10^3
**F**	9.9	0.12	1.5	8.2	0.05	0.3
**Cl**	8.0	0.15	2.8	6.1	0.06	0.6
**Br**	7.3	0.16	3.5	5.4	0.06	0.7
**I**	6.5	0.18	5.0	4.7	0.06	0.8

[a] Computed at ZORA-BP86-D3(BJ)/QZ4P. [b] The orientation of the dihedral angle where each interaction is the most stabilizing (where X = F, Cl, Br, and I). [c] The 3π* unoccupied orbital of the OHC$^\bullet$ fragment and 3σ and 2π occupied orbitals of the $CH_2X^\bullet$ fragment.

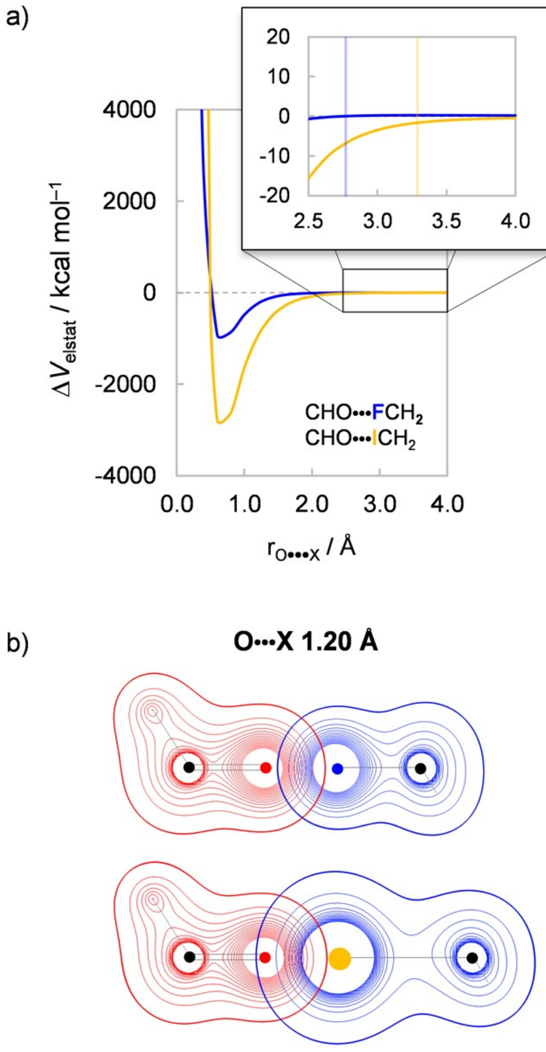

Figure S6. Electrostatic interaction between oxygen and halogen atoms for the C=O•••X–C colinear approach of the OHC$^\bullet$ and $CH_2X^\bullet$ fragments. a) ΔV_{elstat} as a function of the $r_{O\bullet\bullet\bullet X}$ distance (vertical lines indicate $r_{O\bullet\bullet\bullet X}$ separation in the corresponding haloacetaldehyde); b) density contours from –0.9 to 0.9 Bohr^{-3} for OHC$^\bullet$ and $CH_2X^\bullet$ fragments (X = F, I for upper and lower, resp.). Computed at ZORA-BP86-D3(BJ)/QZ4P.

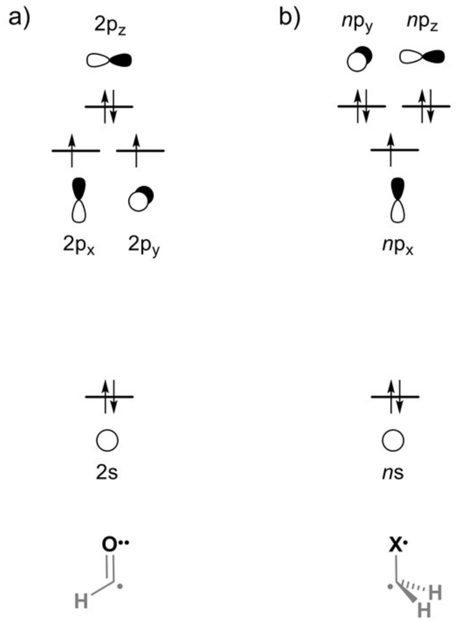

Figure S7. Schematic diagram of the valence configuration of the $O^{••}$ and $X^{•}$ (X = F and I) atoms used for the electrostatic interaction analysis shown in Figure 6c,d in the main text, in which the bare atoms approach each other along the z axis. Note that this reflects the situation for the interaction between the full $OHC^{•}$ and $CH_2X^{•}$ fragments, the parts of which that have been cut away, here, in the $O^{••}$ and $X^{•}$ systems, are shown in faded grey.

Table S4. *Syn* relative to *anti e*lectrostatic interaction $\Delta\Delta V_{elstat}$ energy components (in kcal mol^{-1}) of haloacetaldehydes OHC–CH_2X (X = F, Cl, Br, and I).[a]

X	$\Delta\Delta V_{elstat}$	$\Delta\Delta V_{elstat}\,(n_A n_B)$	$\Delta\Delta V_{elstat}\,(n_A \rho_B)$	$\Delta\Delta V_{elstat}\,(\rho_A n_B)$	$\Delta\Delta V_{elstat}\,(\rho_A \rho_B)$	Δf[b]
**F**	1.2	1,409.1	–1,430.2	–1,468.5	1,490.5	0.2
**Cl**	0.5	2,645.3	–2,667.2	–2,767.4	2,789.8	0.0
**Br**	0.1	5,484.4	–5,510.5	–5,743.0	5,768.9	0.3
**I**	–0.5	8,114.0	–8,135.8	–8,498.8	8,520.0	0.0

[a] Data from the rigid rotation in *syn* geometry but with C–C distance set to 1.51 Å, computed at ZORA-BP86-D3(BJ)/QZ4P. [b] Fitting for incompleteness.

Table S5. Cartesian coordinates (Å), energies (kcal mol^{-1}), and the number of imaginary vibrational frequencies (N_{imag}) of the stationary points in the energy profiles for rotation around the C–C bond of haloacetaldehydes OHC–CH_2X and the open-shell $HOC^{\bullet}$ and $CH_2X^{\bullet}$ fragments (X = F, Cl, Br, and I), computed at ZORA-BP86-D3(BJ)/QZ4P.

***Syn*–fluoroacetaldehyde**
E = -896.9
N_{imag} = 0

C	-0.87690441	-0.08771038	0.00000000
C	0.62587861	0.09020798	0.00000000
O	-1.45765841	-1.14626050	0.00000000
H	-1.42382489	0.89240352	0.00000000
H	0.91093353	0.67739427	0.89136772
H	0.91093353	0.67739427	-0.89136772
F	1.31416093	-1.10645390	0.00000000

***Gauche*–fluoroacetaldehyde**
E = -892.9
N_{imag} = 1, ν = -167.31607i cm^{-1}

C	-0.83525652	-0.19523154	-0.27401587
C	0.59529088	0.04530442	0.22898136
O	-1.55036127	-1.04884085	0.19663180
H	-1.18276050	0.45419916	-1.11445736
H	0.59184615	0.12021427	1.32606517
H	1.03702720	0.94626459	-0.21918065
F	1.38267224	-1.05365595	-0.13669899

***Anti*–fluoroacetaldehyde**
E = -897.8
N_{imag} = 0

C	-4.94984467	-1.64098081	-0.10863772
C	-3.50288152	-1.33117841	0.23137926
O	-5.88093173	-1.15257689	0.49082334
H	-5.08624182	-2.35646893	-0.95724304
H	-3.29567990	-1.62067126	1.27283061
H	-3.31762094	-0.25214257	0.11834504
F	-2.64062963	-2.03109418	-0.61484320

***Syn*–chloroacetaldehyde**
E = -858.5
N_{imag} = 0

C	-0.89048701	-0.08781307	0.00000000
C	0.61399877	0.08395543	0.00000000
O	-1.49392250	-1.13184604	0.00000000
H	-1.42190248	0.89958786	0.00000000
H	0.89847017	0.67147596	0.88511574
H	0.89847017	0.67147596	-0.88511574
Cl	1.53096109	-1.43954515	0.00000000

***Gauche*–chloroacetaldehyde**
E = -856.0
N_{imag} = 1, ν = -171.52758i cm^{-1}

C	-0.85977660	-0.17562469	-0.26403032
C	0.57817177	0.04991713	0.19963510
O	-1.58184633	-1.03633646	0.17886982
H	-1.21935743	0.53736243	-1.04635854
H	0.58281757	0.26623417	1.27497852
H	1.05070798	0.87011732	-0.34937489
Cl	1.57180592	-1.42502341	-0.05623762

***Anticlinal*–chloroacetaldehyde**
E = -859.4
N_{imag} = 0

C	-4.97726201	-1.74866776	0.02288883
C	-3.52267187	-1.39527007	0.28218646
O	-5.88950114	-1.06496911	0.42686150
H	-5.14357656	-2.68946686	-0.55427358
H	-3.19689745	-1.87069238	1.21740838
H	-3.40037454	-0.31141036	0.36473118
Cl	-2.46616463	-2.01151045	-1.03382574

***Anti*–chloroacetaldehyde**
E = -859.3
N_{imag} = 1, ν = -47.48150i cm^{-1}

C	-0.87006059	-0.67406376	0.00000000
C	0.47964884	0.03024507	0.00000000
O	-1.90679217	-0.05202500	0.00000000
H	-0.83574239	-1.78980380	0.00000000
H	0.56105037	0.66516679	0.89004073
H	0.56105037	0.66516679	-0.89004073
Cl	1.84763232	-1.12985756	0.00000000

***Syn*–bromoacetaldehyde**

E = -845.4

N_{imag} = 0

C	-0.89355984	-0.08655038	0.00000000
C	0.60756859	0.08151005	0.00000000
O	-1.50735768	-1.12477373	0.00000000
H	-1.42002774	0.90394928	0.00000000
H	0.90606516	0.65689473	0.88654098
H	0.90606516	0.65689473	-0.88654098
Br	1.60717417	-1.58685813	0.00000000

***Gauche*–bromoacetaldehyde**

E = -843.6

N_{imag} = 1, ν = -164.67148i cm^{-1}

C	-0.87406472	-0.15942520	-0.25271203
C	0.56684379	0.05362639	0.18322610
O	-1.59594312	-1.03134481	0.16939660
H	-1.24607057	0.58912443	-0.99588259
H	0.59856054	0.30584339	1.24940072
H	1.06008752	0.83133735	-0.40539462
Br	1.62809501	-1.58419724	-0.02182747

***Anticlinal*–bromoacetaldehyde**

E = -846.8

N_{imag} = 0

C	-4.92579432	-1.82385935	0.15807148
C	-3.47990248	-1.46180002	0.40231809
O	-5.82950487	-1.02218082	0.24362879
H	-5.10583387	-2.89097761	-0.11381579
H	-3.05882524	-2.03205314	1.23807981
H	-3.34990902	-0.38686018	0.54263845
Br	-2.45214514	-2.00321050	-1.18939026

***Anti*–bromoacetaldehyde**

E = -846.1

N_{imag} = 1, ν = -74.16080i cm^{-1}

C	-0.97388205	-0.66362509	0.00000000
C	0.40854757	-0.03197945	0.00000000
O	-1.97117871	0.02061851	0.00000000
H	-1.00996083	-1.77872742	0.00000000
H	0.53703497	0.59054340	0.89171806
H	0.53703497	0.59054340	-0.89171806
Br	1.83754363	-1.37357779	0.00000000

***Syn*–iodoacetaldehyde**

E = -834.3

$\boldsymbol{N_{imag}}$ = 0

C	-0.89843142	-0.08167042	0.00000000
C	0.60060356	0.08253120	0.00000000
O	-1.51782827	-1.11737900	0.00000000
H	-1.42552666	0.90826164	0.00000000
H	0.90487355	0.65606489	0.88531528
H	0.90487355	0.65606489	-0.88531528
I	1.70406106	-1.76113128	0.00000000

***Gauche*–iodoacetaldehyde**

E = -833.1

$\boldsymbol{N_{imag}}$ = 1, ν = -150.57024i cm^{-1}

C	-0.88838105	-0.13840872	-0.23683699
C	0.55949314	0.05895731	0.16029294
O	-1.60495475	-1.02840720	0.15751175
H	-1.28031275	0.65202063	-0.92524514
H	0.61763507	0.33910380	1.21868848
H	1.05415582	0.81513119	-0.45424644
I	1.71792178	-1.76146203	-0.01133764

***Anticlinal*–iodoacetaldehyde**

E = -836.3

$\boldsymbol{N_{imag}}$ = 0

C	-4.89438232	-1.84464147	0.20350702
C	-3.45373463	-1.48938404	0.45002462
O	-5.78258384	-1.02086707	0.15222154
H	-5.09454063	-2.93366555	0.06148973
H	-3.00589221	-2.09129461	1.24719671
H	-3.31165081	-0.41853993	0.60680989
I	-2.36120792	-2.04613549	-1.34899724

***Anti*–iodoacetaldehyde**

E = -834.5

$\boldsymbol{N_{imag}}$ = 1, ν = -103.08701i cm^{-1}

C	-1.09541926	-0.77428604	-0.13961112
C	0.23725323	-0.39169652	0.48268382
O	-2.08516465	-0.10888022	0.06203532
H	-1.11069482	-1.68662291	-0.78163092
H	0.15613277	-0.39286790	1.57457863
H	0.53327413	0.60901005	0.15134527
I	1.83494980	-1.74859412	-0.04775879

Formyl radical

E = -393.8

N_{imag} = 0

C	0.14701429	-0.33290388	0.00000000
H	0.88369536	0.52960719	0.00000000
O	-1.02716672	-0.20079752	0.00000000

Fluoromethyl radical

E = -421.4

N_{imag} = 0

H	0.07972884	0.56770797	0.96643106
C	-0.09663861	0.10262757	0.00000000
H	0.07972884	0.56770797	-0.96643106
F	-0.05927603	-1.24536088	0.00000000

Chloromethyl radical

E = -385.1

N_{imag} = 0

H	-0.06684151	0.56291984	0.95859566
C	-0.06321473	0.05778043	0.00000000
H	-0.06684151	0.56291984	-0.95859566
Cl	-0.05100973	-1.64213383	0.00000000

Bromomethyl radical

E = -370.3

N_{imag} = 0

H	-0.06707942	0.54053923	0.96024707
C	-0.06418621	0.03981111	0.00000000
H	-0.06707942	0.54053923	-0.96024707
Br	-0.04461256	-1.81882173	0.00000000

Iodomethyl radical

E = -358.7

N_{imag} = 0

H	-0.07157004	0.53622196	0.95538321
C	-0.06340755	0.02512446	0.00000000
H	-0.07157004	0.53622196	-0.95538321
I	-0.03871005	-2.02156433	0.00000000
